# Analysis of Transcriptional Responses of the Inflorescence Meristems in *Jatropha* *curcas* Following Gibberellin Treatment

**DOI:** 10.3390/ijms19020432

**Published:** 2018-02-01

**Authors:** Wen-Kai Hui, Yi Wang, Xiao-Yang Chen, Mohamed Zaky Zayed, Guo-Jiang Wu

**Affiliations:** 1 National Engineering Laboratory for Forest Tree Breeding, College of Biological Science and Technology, Beijing Forestry University, Beijing 100083, China; xwk168@126.com; 2 State Key Laboratory for Conservation and Utilization of Subtropical Agro-Bioresources, Guangdong Key Laboratory for Innovative Development and Utilization of Forest Plant Germplasm, College of Forestry and Landscape Architecture, South China Agricultural University, Guangzhou 510642, China; x_wangyier@163.com (Y.W.); mzmohamedzaky86@gmail.com (M.Z.Z.); 3 Forestry and Wood Technology Department, Faculty of Agriculture (EL-Shatby), Alexandria University, Alexandria 21527, Egypt; 4 Key Laboratory of Plant Resources Conservation and Sustainable Utilization, South China Botanical Garden, Chinese Academy of Sciences, Guangzhou 510650, China

**Keywords:** *Jatropha curcas* L., gibberellin, transcriptome, inflorescence meristems, DEGs

## Abstract

*Jatropha curcas* L. seeds an oilseed plant with great potential for biodiesel production. However, low seed yield, which was limited by its lower female flowers, was a major drawback for its utilization. Our previous study found that the flower number and female-to-male ratio were increased by gibberellin treatment. Here, we compared the transcriptomic profiles of inflorescence meristem at different time points after gibberellic acid A3 (GA_3_) treatment. The present study showed that 951 differentially expressed genes were obtained in response to gibberellin treatment, compared with control samples. The 6-h time point was an important phase in the response to exogenous gibberellin. Furthermore, the plant endogenous gibberellin, auxin, ethylene, abscisic acid, and brassinolide-signaling transduction pathways were repressed, whereas the genes associated with cytokinin and jasmonic acid signaling were upregulated for 24-h time point following GA_3_ treatment. In addition, the floral meristem determinacy genes (*JcLFY*, *JcSOC1*) and floral organ identity genes (*JcAP3*, *JcPI*, *JcSEP1-3*) were significantly upregulated, but their negative regulator (*JcSVP*) was downregulated after GA_3_ treatment. Moreover, the effects of phytohormone, which was induced by exogenous plant growth regulator, mainly acted on the female floral differentiation process. To the best of our knowledge, this data is the first comprehensive analysis of the underlying transcriptional response mechanism of floral differentiation following GA_3_ treatment in *J. curcas*, which helps in engineering high-yielding varieties of *Jatropha*.

## 1. Introduction

*Jatropha curcas* L. (Euphorbiaceae) is a native species of South America [1]. Depending on geographic and climatic conditions, up to 60% of oil in *Jatropha* seeds can be used directly or in transesterified form as a biodiesel [2,3]. However, its utilization as bio-energy plant was limited by its low seed yield, which was caused by low number female flowers [4,5]. Thus, seeking a useful way to improve the fruit yield and uncovering the underlying reasons associated with floral differentiation in *J. curcas*, especially female floral differentiation, were critical for the improvement of the cultivation of high-yielding *J. curcas* germplasms.

Recently, researchers reported that the fruit yield of *J. curcas* could be improved by applying plant growth regulators [4,5,6,7]. Our previous study found that spraying gibberellin acid A3 (GA_3_) onto the inflorescence meristems of *J. curcas* could significantly increase the number of female and male flowers and the female-to-male flower ratio, which was benefit to an increase in fruit yield [8]. Gibberellins (GAs) play a major role in the network of floral induction pathways [9], it also acts very important regulated roles in the female flower differentiation process [10]. In GAs biosynthesis and catabolism pathways, the bioactive GAs, including GA_1_ and GA_4_, could be catalyzed by GA 20-oxidases (GA20OX) and GA 3-oxidases (GA3OX), but the bioactive GAs can be converted to inactive forms by GA 2-oxidases (GA2OX) [11]. The number of flowers could be decreased by the overexpression of *JcGA2OX6* in *J. curcas* [12].DELLA proteins were nuclear negative regulators to repress GA signaling. The GAs receptor, GID1 (GA Insensitive Dwarf 1), could achieve GAs signaling transduction by degrading DELLA proteins [13]. The fruit yield was also increased by exogenous GAs treatment in some species [14,15]. Furthermore, the exogenous GAs treatment could regulate the expression of DELLA proteins, GID1, and GA oxidases to influence the endogenous GA biosynthesis and signaling transduction pathways [16]. To date, these key genes related to the endogenous GA biosynthesis and signaling transduction pathways were few described in *J. curcas*.

Moreover, phytohormones showed complex interaction networks to regulate the floral differentiation process. IAA (Auxin) plays a major role in multiple biosynthesis pathways regulating floral development [17]. CTK (Cytokinin) was known to contribute to grain yield by affecting the source/sink transition in rice and *J. curcas* [18,19], and ABA (Abscisic acid) signaling transduction pathway could be repressed by exogenous CTK treatment in *J. curcas* [19]. JA (Jasmonic acid) is required for pollen maturation, which could be blocked without JA biosynthesis in *Arabidopsis* [20].The exogenous GAs treatment not only affected the endogenous GAs signaling, but also influenced the other phytohormones-signaling transduction pathways, and their co-regulated factor was the DELLA proteins [21]. In addition, GA could regulate the floral differentiation by interacting with MADS-box transcription factors, which play vital regulatory roles in the floral organ differentiation process [16]. There were 107 members related to MADS-box genes, which were clustered into class A, B, C, D and E to co-determine the fate of floral organ primordium by the complex crosstalk network [22]. GAs signaling could promote the expression of *FLOWERING LOCUS TIME* (*FT*), *SUPPRESSOR OF OVEREXPRESSION OF CONSTANS 1* (*SOC1*) and *LEAFY* (*LFY*) in inflorescence meristem to stimulate early flowering in *Arabidopsis* [23,24,25]. The MADS-box transcription factor *SHORT VEGETATIVE PHASE* (*SVP*), a critical inhibitor of flowering, could delay flowering by repressing GA biosynthesis [26]. A cucumber DELLA homolog *CsGAIP* could decrease the transcription of B class homeotic genes *APETALA3* (*AP3*) and *PISTILLATA* (*PI*) to inhibit floral development in *Arabidopsis* [27]. However, the information regarding large-scale transcriptome alterations in response to exogenous GAs application in *J. curcas* remains scarce.

Therefore, the present study was conducted to compare the transcriptomic profiles of inflorescence meristems in different time points after gibberellin treatment using Illumina Hiseq 4000 platform to uncover the underlying transcriptional response mechanism of floral differentiation in *J. curcas*. The significant enrichment metabolic pathways related to floral differentiation, differentially expressed genes (DEGs) involved in the biosynthesis and signaling of gibberellins and the other phytohormones, and MADS-box transcription factors after GA_3_ treatment were also identified. This study will provide insights for determining the mechanism of gibberellins action on floral differentiation in *J. curcas*.

## 2. Results

### 2.1. Morphological Observation after GA_3_ Treatment

**Figure 1:**
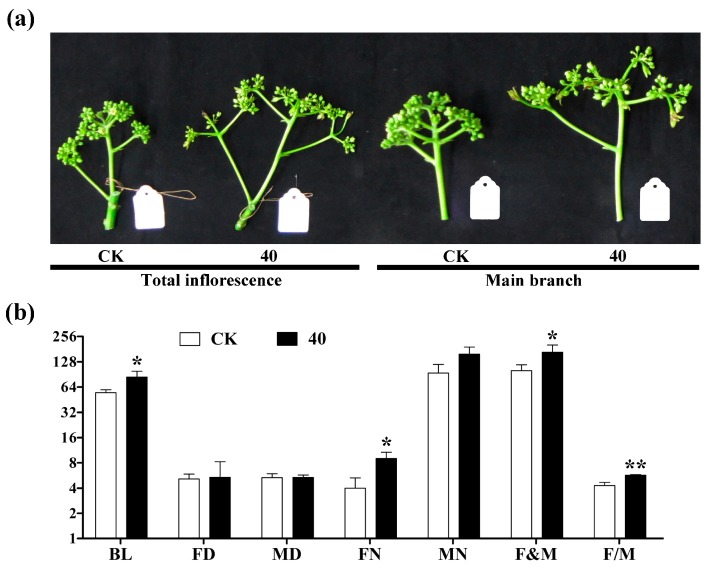
The morphology and statistics of the investigated traits after GA_3_ treatment in *J. curcas*. (**a**) the morphology of the inflorescence after GA_3_ treatment at 22 days, CK (control check) was treated by distilled water and 40 was treated by 40 mg/L GA_3_; (**b**) the statistics of the investigated traits after GA_3_ treatment at 22 days, BL was the length of the inflorescence, FD was the diameter of female flower, MD was the diameter of male flower, FN was the number of female flower, MN was the number of male flower, F&M was the total number of female and male flowers, F/M was the ratio of female to male flowers. The error bar means S.D., which was calculated by fifteen inflorescence meristems after the GA_3_ and control treatment, respectively. * is significant difference (*p* < 0.05), ** is very significant difference (*p* < 0.01).

In order to ascertain the effects of 40 mg/L GA_3_ treatment on inflorescence meristems (about 0.5 cm in diameter), the morphological analyses were conducted on *Jatropha* inflorescence treated with working solution bearing or lacking GA_3_, respectively. The length of inflorescence (BL) was significantly increased at 22 days after GA_3_ treatment, whereas the diameters of female and male flower (FD and MD) were not changed (Figure 1). Moreover, the female floral number (FN), total floral number (F&M), and the ratio of female to male flowers (F/M) were significantly improved after 40 mg/L GA_3_ treatment. In addition, more male flowers were also induced with no significant difference (*p* > 0.05). These results indicated that the floral differentiation could be promoted by exogenous gibberellins application in *J. curcas*, especially female flowers.

### 2.2. Illumina Sequencing of Different cDNA Libraries

After the quality control, 6.27–10.46 G clean reads were produced from eighteen cDNA libraries in this study, respectively (Table S1). The error rate of RNA-seq was only 0.01%, all of the Q30 (Phred score) were more than 93%, and the GC content was more than 42% in each sample. In addition, when mapped to the genome data of *J. curcas*, the alignable reads ranged from 54.39 million (T24_2, 88.16%) to 36.15 million (T6_1, 86.50%) based on clean reads (Table S2). Among the mapped reads, 35.36–53.29 million were uniquely aligned reads, with T24_2 making up the highest percentage (86.39%). Moreover, 8532 novel transcripts were found in present RNA-seq analysis, which could contribute to obtain new genes and exons related to inflorescence meristem differentiation in *J. curcas*.

### 2.3. DEGs Annotation and Enrichment in Response to GA_3_ Treatment

**Figure 2:**
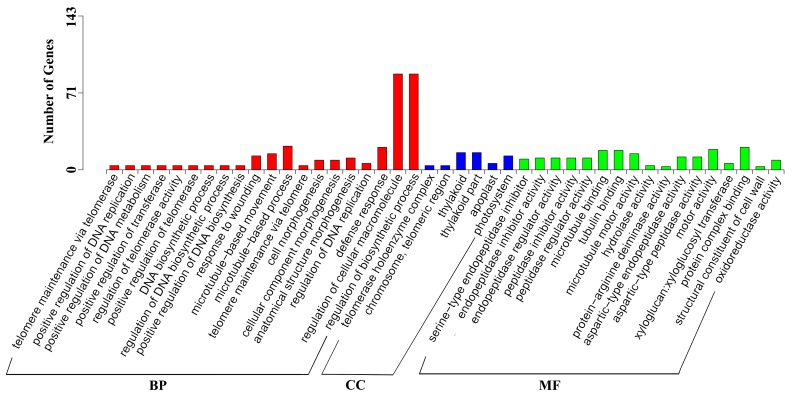
Gene ontology categories of DEGs after GA_3_ treatment in *J. curcas*. BP: biological process, CC: cellular component, MF: molecular function, and *p* < 0.05 is the significant enrichment level.

**Figure 3:**
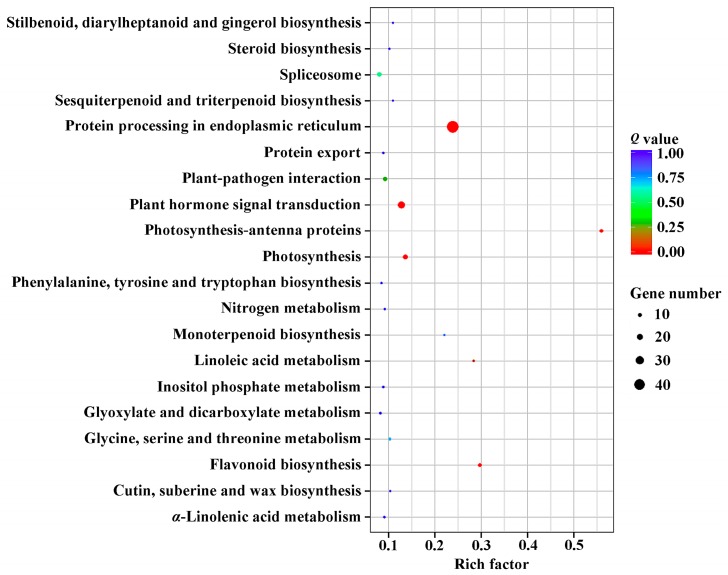
The statistics of Kyoto Encyclopedia of Genes and Genomes (KEGG) pathway enrichment involved in DEGs after GA_3_ treatment in *J. curcas*. The Rich factor indicated the percentages of DEGs belong to the corresponding pathway. The sizes of bubble represent the number of DEGs in the corresponding pathway, and the colors of the bubble represent the enrichment *Q* value of the corresponding pathway.

The DEGs were detected in four comparisons, T6 vs. CK6, T12 vs. CK12, T24 vs. CK24, and T48 vs. CK48, to perform the transcriptional response analysis of the inflorescence meristems in *J. curcas*. In total, 951 DEGs were obtained from these four comparisons, and these DEGs were annotated to 43 orthologous terms (Figure 2), including 20 terms involved in biological processes that were mainly associated with DNA replication and macromolecules biosynthesis process, 6 terms involved in cellular components that were significantly annotated to telomerase and thylakoid region, and 17 terms involved in molecular functions that were mainly related to the activity of peptidase and microtubule protein. Furthermore, the 951 DEGs were significantly enriched in protein processing in the endoplasmic reticulum, plant hormone signal transduction, photosynthesis, and flavonoid biosynthesis pathways (Figure 3). It indicated that the more actively biological synthesis and metabolism was showed in *Jatropha* inflorescence after GA_3_ treatment.

**Figure 4:**
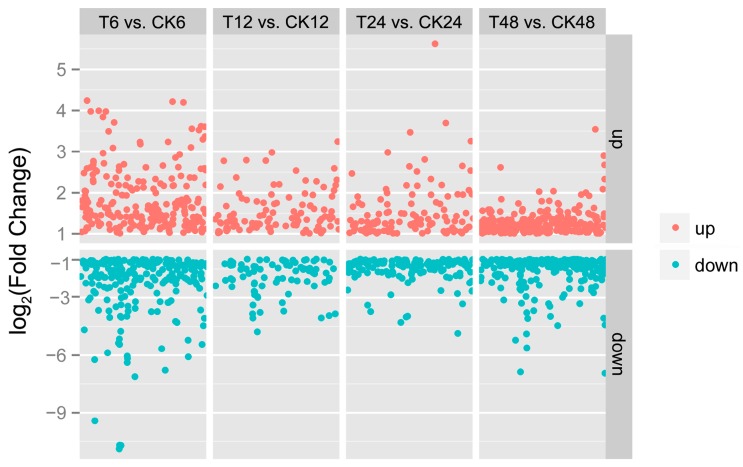
Distribution of the DEGs at different time points after GA_3_ treatment in *J. curcas*. The selected level of the DEGs was |log_2_(Fold Change)| > 1.

In addition, almost equal DEGs number was obtained between up- and down-regulation at each time point (Figure 4). However, the greatest overall number and larger fold change of DEGs were obtained at 6-h time point. Furthermore, GO (Gene Ontology) enrichment showed that the number of DEGs in each category, such as “metabolic process”, “catalytic activity”, “organic substance metabolic process”, “cellular process”, and “primary metabolic process”, were highest at the 6-h time point (Figure S1).All of these results indicated that 6-h time point may be an important phase in the response to exogenous gibberellins in *J. curcas*.

**Figure 5:**
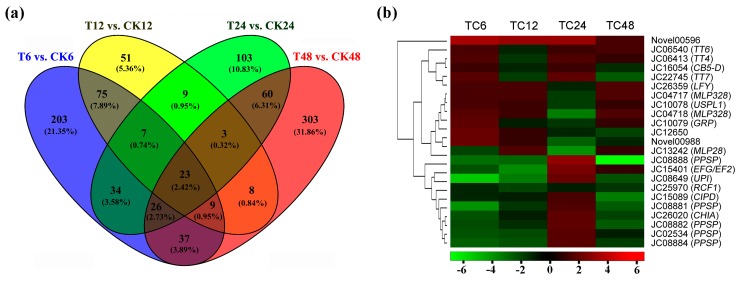
In figure body the DEGs analysis of different time points after GA_3_ treatment in *J. curcas*. (**a**) venny diagram of the DEGs detected in each comparison; (**b**) the heat-map of the DEGs co-detected in all of the comparisons, the color was involved in the log_2_(Fold change) of DEGs in different comparison, red was upregulation and green is downregulation. In addition, TC6: T6 vs. CK6, TC12: T12 vs. CK12, TC24: T24 vs. CK24, TC48: T48 vs. CK48, and the same below.

Moreover, there were 23 DEGs co-detected in the four comparisons (Figure 5a). Three DEGs of them, JC12650, Novel00988, and Novel00596 (Novel means a new transcript) were upregulated at 6 and 12-h time points, and the Novel00596 was still upregulated in the following time points (Figure 5b), but no homologous sequence of these three genes was isolated in TAIR10 protein database (Table 1), which suggested that these sequences may be some specific genes involved in response to GA_3_ treatment in *Jatropha* inflorescence meristems. Furthermore, JC06413 (*JcTT4*), JC06540 (*JcTT6*), and JC22745 (*JcTT7*) were annotated to flavonoid biosynthetic process, and upregulated at 6-h and 12-h time points, respectively. An important transcription factor associated with the transitions from vegetative to reproductive growth, *JcLFY* (JC26359), was identified in these 23 DEGs library, and it was also upregulatedat 6-h and 12-h time points. Interestingly, eight DEGs were only significantly upregulated at 24-h time point after GA_3_ treatment, JC02534, JC08649, JC08881, JC08882, JC08884, JC08888, JC15089, and JC26020 (Figure 5b). The Blastx with TAIR10 protein database showed that these genes were mainly related to phosphorylase superfamily proteins, which indicated that 24-h point may be the other important phase in the response to exogenous gibberellins.

### 2.4. DEGs Involved in Gibberellin Biosynthesis, Metabolism and Signaling

**Figure 6:**
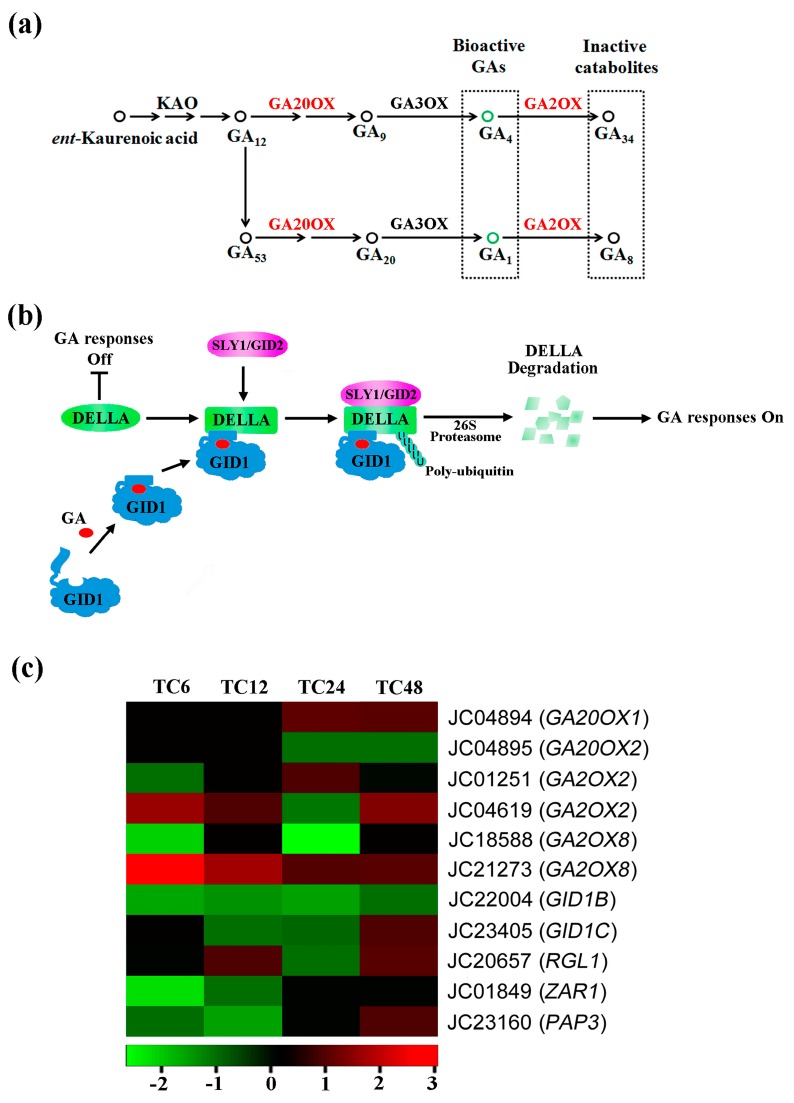
The DEGs analysis associated with gibberellin biosynthetic and signaling pathways after GA_3_ treatment in *J. curcas*. (**a**) Gibberellin biosynthetic pathway, KAO: *ent*-kaurenoic acid oxidase, GA20OX: GA 20-oxidase, GA2OX: GA 2-oxidase, GA3OX: GA 3-oxidase; (**b**) Gibberellin-signaling transduction pathway, T bar is the inhibition; (**c**) The heat-map of DEGs associated with GA biosynthesis and signaling pathways after GA_3_ treatment.

Based on the above analysis, the DEGs library was significantly enriched in plant hormone signal transduction pathways (Figure 3). In order to obtain more information about these details, 12 DEGs were identified to perform the further analysis involved in GA biosynthesis, metabolism and signaling pathways. Based on the Blastx with TAIR10 protein database, two DEGs related to bioactive GA formation (Figure 6a, Table 2), JC04894 (*JcGA20OX1*) and JC04895 (*JcGA20OX2*), were not shown differential expression until 24-h time point after GA_3_ treatment (Figure 6c). However, the DEGs encoding GA-inactivating enzymes, *JcGA2OX2* and *JcGA2OX8,* were upregulated at different time points after GA_3_ treatment, especially JC21273 (*JcGA2OX8*). It indicated that the endogenous gibberellin production was reduced by exogenous GA_3_ application in *J. curcas*.

The GA-GID1-DELLA complex was mediated via the binding of GA to GID1, which caused a rapid degradation of DELLAs through the ubiquitin-proteasome pathway (Figure 6b). The SLY1/GID2, a specific ubiquitin E3 ligase complex, was required to recruit DELLA protein for the subsequent degradation by the 26S proteasome. After that, the GA signaling could be transmitted into the following pathways. In the present study, two *Jatropha* ortholog genes encoding a GA receptor, JC22004 (*JcGID1B*) and JC23405 (*JcGID1C*), were screened in the DEGs dataset after GA_3_ treatment (Table 2). Furthermore, *JcGID1B* was downregulated at each time point following GA_3_ treatment, while the *JcGID1C* was not shown differential expression in T6 vs. CK6 and significantly downregulated at the 12 and 24-h time points (Figure 6c). Additionally, two DEGs involved in GA signal transduction pathways, JC01849 (*JcZAR1*) and JC23160 (*JcPAP3*), were also downregulated after GA_3_ treatment. Meanwhile, JC20657 (*JcRGL1*), a gene encoding the DELLA protein, was upregulated at 12 and 48-h time points after GA_3_ application. These results suggested that exogenous GA_3_ reduced the endogenous gibberellin activity by downregulating GA receptor genes and upregulating suppressors of GA signaling.

### 2.5. Gibberellin-Regulated DEGs Involved in the Signaling of Other Phytohormones

**Figure 7:**
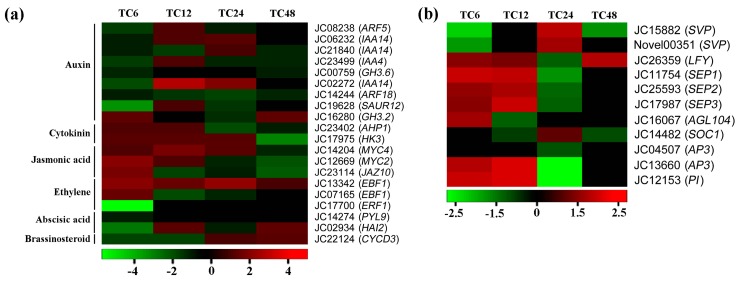
The heat-map of the DEGs related to the other plant hormone-signaling transduction pathways and MADS-box transcription factors after GA_3_ treatment in *J. curcas*. (**a**)The heat-map of the DEGs involved in the other plant hormone-signaling transduction pathways; (**b**) the heat-map of the DEGs associated with MADS-box transcription factor. The color was log_2_(Fold change) of the DEGs in different comparison, red is upregulated and green is down regulated.

Signaling pathways of phytohormones showed complex interaction networks to regulate floral differentiation in plants. In order to understand the roles of other phytohormones in inflorescence meristems in response to GA application, we identified homologous genes related to various hormonal transduction pathways in the 951 DEGs library by Blastx with TAIR10 database. In total, 20 DEGs were obtained to conduct the further analysis associated with auxin (IAA), cytokinin (CTK), jasmonic acid (JA), ethylene (ETH), abscisic acid (ABA), and brassinosteroid (BR)-signaling transduction pathways (Table 3). Nine DEGs were involved in auxin-activated signaling pathway (Table 3), and eight of them: JC08238 (*JcARF5*), JC06232 (*JcIAA14*), JC21840 (*JcIAA14*), JC23499 (*JcIAA4*), JC00759 (*JcGH3.6*), JC02272 (*JcIAA14*), JC14244 (*JcARF18*), and JC19628 (*JcSAUR12*) were significantly downregulated at 6 and 48-h time points after GA_3_ treatment (Figure 7a), whereas only JC16280 (*JcGH3.2*), a downstream gene of IAA-signaling pathway, was significantly upregulated at 6 and 48-h time points. It indicated that the antagonistic interactions were existed between GA and IAA in the development of inflorescence meristems in *J. curcas*.

Two DEGs, JC23402 (*JcAHP1*) and JC17975 (*JcHK3*), were involved in cytokinin-activated signaling pathway (Table 3), and both of them were upregulated at 6 and 12-h time points (Figure 7a). *JcAHP1*, a gene encoding the histidine phosphotransfer protein 1, was the vital factor related to the CTK signaling transduction from cytoplasm to nucleus. *JcHK3*, a gene encoding the histidine kinases, acted as the cytokinin sensor in CTK signaling pathways. The upregulation of these genes could significantly enhance the cytokinin signaling after GA_3_ application.

Meanwhile, three DEGs, JC14204 (*JcMYC4*), JC12669 (*JcMYC2*), and JC23114 (*JcJAZ10*), were associated with jasmonic acid-signaling pathway. Similarly with the CTK signaling after GA_3_ application, JC14204 (*JcMYC4*) and JC12669 (*JcMYC2*) were upregulated at 6 and 12-h time points (Figure 7a), which were related to Jasmonic acid-activated signaling pathway (Table 3). However, JC23114 (*JcJAZ10*), a gene encoding jasmonate ZIM-domain (JAZ) proteins, which was a key negative regulator of JA signaling, was upregulated at 6-h time point after GA_3_ treatment. These results indicated that the intricate agonistic interactions were shown between GA and JA in the differentiation of inflorescence meristems in *J. curcas*. The exogenous gibberellin could induce the endogenous jasmonicacid-signaling pathway within a certain range.

Based on the Blastx with TAIR10 database, three DEGs were related to ethylene-signaling transduction pathway (Figure 7a). JC13342 and JC07165 were annotated to *Jatropha* orthologs of *EBF1*, which was involved in the negative regulation of ethylene-activated signaling pathway (Table 3). Both of them were upregulated at 6-h time point after GA_3_ treatment (Figure 7a). Furthermore, JC17700 (*JcERF1*), a key ethylene-responsive transcription factor, was significantly downregulatedat 6-h time point, and then it was not detected differential expression at the following time points after GA_3_ treatment. These results indicated that a negative crosstalk was shown between GA and ETH-signaling pathways in *J. curcas*.

Moreover, two DEGs, JC14274 (*JcPYL9*) and JC02934 (*JcHAI2*), were involved in abscisic acid-signaling transduction pathway (Table 3). JC14274 (*JcPYL9*), an ABA receptor belongs to *PYR1-like*, was associated with abscisic acid-activated signaling pathway. JC02934 (*JcHAI2*), a gene encoding the protein phosphatase 2C, was a negative regulator of ABA signaling. Both of them were downregulated at 6-h time point after GA_3_ treatment (Figure 7a), whereas JC02934 (*JcHAI2*) was significantly upregulated at 12 and 48-h time points. In addition, one DEG, JC22124 (*JcCYCD3*), was detected and annotated to brassinosteroid-signaling transduction pathway. It was downregulated at 6 and 12-h time points after GA_3_ application (Figure 7a).These results implied that ABA and BR signaling was repressed in the inflorescence differentiation process of *J. curcas* after exogenous GA_3_ treatment.

### 2.6. MADS-Box Transcription Factor in Response to GA_3_ Treatment

Gibberellin could regulate the floral differentiation by interacting with MADS-box transcription factors, which play vital regulatory roles in the floral organ differentiation process. 11 differentially expressed GA-responsive transcription factors were identified in the present study (Table 4).

Based on the Blastx with TAIR10 database, two DEGs, JC15882 and Novel00351, were annotated to *Jatropha* orthologous *SVP*, a negative regulator of flowering. Both of them were downregulated at 6 and 48-h time points after GA_3_ treatment (Figure 7b). However, JC26359 (*JcLFY*), a positive regulator of flowering, was significantly upregulated at 6, 12, and 48-h time points. Meanwhile, *JcSOC1* (JC14482), an upstream gene of *JcLFY*, was also upregulated at 24-h time point after GA_3_ treatment. It indicated that the flowering time could be accelerated by exogenous GA_3_ treatment in *J. curcas*, which was consistent with the previous studies [28,29].

Additionally, floral organ identity genes played important roles in floral sex differentiation process. In our previous study, ABCDE model was formulated to regulate the floral organ identification in *J. curcas* [30]. In this study, 3 DEGs of E-function, JC11754, JC25593 and JC17987, were isolated and annotated to *JcSEP1*, *JcSEP2*, and *JcSEP3*, respectively (Table 4). All of them were upregulated at 6 and 12-h time points, whereas they were downregulated at 24-h time point (Figure 7b). Moreover, 3 DEGs of B-function, JC04507 (*JcAP3*), JC13660 (*JcAP3*), and JC12153 (*JcPI*) were identified in the 951 DEGs library. Similarly with the DEGs in class E, JC13660 and JC12153 were significantly upregulated at 6 and 12-h time points and downregulated at 24-h time point (Figure 7b). It indicated that exogenous GA could promote the B- and E-function transcription factor, which might be a reason that the floral number was increased after GA_3_ treatment.

### 2.7. DEGs Involved in Male and Female Floral Differentiation

In order to obtain more information about the DEGs involved in male and female floral differentiation after GA_3_ treatment, the 951 DEGs detected in the present study was combined with our previous transcriptome dataset (Gene Expression Omnibus number: GSE102894) related to floral sex differentiation process [30], including male floral initiation stage (STD1 vs. IND, IND was the stage of inflorescence meristems, and STD1 was the stage of male floral initiation), male floral development stage (STD2 vs. STD1, STD2 was the stage of ten complete stamens formed), female floral initiation stage (PID1 vs. IND, PID1 was the stage of female floral initiation), and female floral development stage (PID2 vs. PID1, PID2 was the stage of complete carpel and ovary formed).

The results showed that 55 DEGs were isolated and associated with male floral initiation after GA_3_ application (Figure S2). Four DEGs of them (JC11710, JC18282, JC20688, JC21298) were significantly annotated to flavonoid biosynthetic process by KEGG enrichment analysis (Figure S3a). Interestingly, these DEGs were mainly upregulated both in T6 vs. CK6 and STD1 vs. IND. Moreover, 32 DEGs were co-detected in the DEGs libraries of GA_3_ treatment and STD1 vs. IND and STD2 vs. STD1, and 54 DEGs were identified both in the DEGs libraries of GA_3_ treatment and STD2 vs. STD1 (Figure S2). KEGG enrichment showed that these genes were mainly involved in DNA replication, protein biosynthesis, and sugar metabolism pathways (Figure S3b), indicating that these genes were contributed to the male flower development after GA_3_ treatment.

There were 16 DEGs co-detected in the DEGs libraries of GA_3_ treatment and PID1 vs. IND (Figure S2). Based on the KEGG enrichment analysis (Figure S4a), two DEGs of them, JC23402 (*JcAHP1*) and JC04692 (*JcPIN3*), were significantly associated with CTK and IAA signal transduction pathways, respectively (Table 3). In addition, eighteen DEGs were isolated both in the DEGs libraries of GA_3_ treatment and PID1 vs. IND and PID2 vs. PID1, while one hundred and ten DEGs were obtained in the DEGs libraries of GA_3_ treatment and PID2 vs. PID1. The number of these DEGs was about 1.5 times of the corresponding period in male floral differentiation process (Figure S2). KEGG enrichment showed that these 128 DEGs were significantly related to plant hormone-signaling transduction pathways (Figure S4b). It indicated that the female floral differentiation was significantly involved in plant hormone-signaling transduction pathways after exogenous GA_3_ treatment.

### 2.8. qRT-PCR Validation

**Figure 8:**
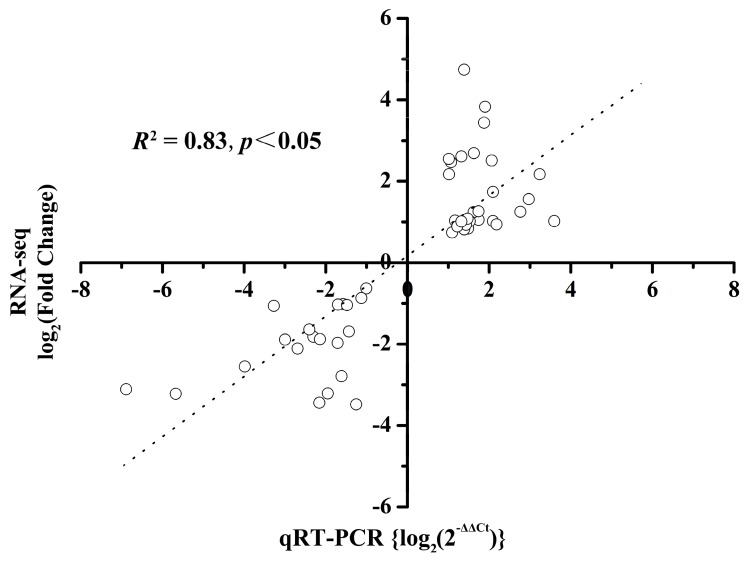
The correlation between qRT-PCR and RNA-seq data. Correlation between qRT-PCR and RNA-seq data of 48 selected DEGs: 28 upregulated genes and 20 downregulated genes in 4 pairs of amplified RNA samples. The dashed line was the fitting curve estimated by correlation analysis, and Spearman Rank Correlation coefficient = 0.83 (*p* < 0.05).

To validate the RNA-seq data, 48 DEGs detected by T6 vs. CK6, T12 vs. CK12, T24 vs. CK24, and T48 vs. CK48 were tested by qRT-PCR (Table S3). These genes were selected because of their important function identified in this study, including 20 downregulated genes and 28 upregulated genes. All of them were consistent with the same trend of upregulation or downregulation between the two different expression analysis platforms (Figure 8). The correlation of the two expression measurements was 0.83 between these 48 genes (*R*^2^ = 0.83). In a word, the results of RNA-seq and qRT-PCR were consistent.

## 3. Discussion

GA signaling is one of the important regulated factors in the network of floral induction pathways [9]. Recently, Chen et al. [31] found that the exogenous application of GA_3_ could partially prevent pistil development to generate neutral flowers without stamens and pistils in gynoecious *Jatropha*. However, Makwana et al. [18] found that the female flower and fruit yield could be increased by exogenous GA_3_ in wild *J. curcas*. Our previous study was consistent with the Makwana’s research [8]. From the present study, the female flower number and female-to-male flower ratio were significantly increased after GA_3_ treatment (Figure 1). Therefore, we supposed that whether the application of exogenous GA_3_ to *J. curcas* inflorescences stimulates floral differentiation may be cultivar-dependent.

To advance our understanding of underlying GA-induced responses in *J. curcas*, the transcriptome analysis of *Jatropha* inflorescence meristems were carried out after GA_3_ treatment. We also performed the control samples at the same conditions to remove some genes that were differential expression following the plant growth, but not the GA_3_ treatment between different time points. Moreover, different plant species were given specific response times for various plant growth regulators [32]. In the previous study, GA content of the berry was substantially increased for 24 h following GA_3_ application in grapevine flower [33]. Chen et al. [34] found that the DEGs were significantly increased at 12-h time point after exogenous cytokinin treatment in *J. curcas*. In order to determine the appropriate time point in the present study, we collected the five time point samples from 0 h to 48 h after 40 mg/L GA_3_ treatment in *J. curcas*. Fortunately, 6.27–10.46 G clean data were obtained in GA_3_ treated and untreated samples, respectively. 951 DEGs were isolated at the different time points, compared with control samples. In addition, 8532 novel transcripts were found in present RNA-seq analysis, which could contribute to obtain new genes and exons related to inflorescence meristem differentiation in *J. curcas*. Furthermore, we supported that 6-h time point was an important phase in the response to exogenous gibberellin in this study.

Gibberellins regulate various aspects of plant growth and development [35], it also play a major role in the network of floral induction pathways [36]. In the present study, the DEGs annotated to GAs biosynthesis and signaling were further analyzed. We found that *GA20OX1* (JC04894) and *GA20OX2* (JC04895), which catalyzed the formation of bioactive GAs [37], were not detected differential expression until 24-h time points after GA_3_ treatment (Figure 6c). In contrast, the genes encoding GA2OX, a major GA inactive catabolic oxidase [11], were upregulated after GA_3_ treatment. It indicated that exogenous GA could inhibit the endogenous GA biosynthesis process. This finding was consistent well with previous studies [33,38]. Therefore, we suggested that the content of bioactive GAs might show a feedback regulation to response exogenous GA_3_ application in *J. curcas*. The other evidence supporting this suggestion was that the GA receptor genes, *JcGID1B* (JC22004) and *JcGID1C* (JC23405), were significantly downregulated at 6 and 24-h time points after GA_3_ application in the present study, but *JcRGL1* (JC20657), a gene encoding DELLA protein (Figure 6b,c), was upregulated at 12-h time point, which agreed with the previous researches [39,40]. In further study, a strategic approach is to investigate the concentration of endogenous gibberellins after exogenous GA_3_ treatment in *J. curcas*. 

In addition, gibberellins could jointly regulate the floral differentiation by crosstalk with various phytohormone-signaling pathways in *J. curcas*. In previous study, the exogenous CTK application could significantly downregulate the expression of the genes involved in ABA, and ETH signal transduction pathways in *J. curcas* [19]. The similar results in the present study were that the ABA, ETH, and BR signaling were also repressed after exogenous GA treatment. It indicated that these phytohormone-signalings were not important for floral differentiation process in *J. curcas*. Moreover, we detected that the antagonistic interactions were existed between GA and IAA in the development of inflorescence meristems in *J. curcas*. That might be the underlying reason why the female flowers were induced by spraying exogenous GA onto the inflorescence, but the highest concentration of GA resulted in the withering of the *Jatropha* inflorescence in previous studies [8,18].

However, the DEGs involved in the JA-signaling transduction pathway, such as *JcMYC2* (JC12669) and *JcMYC4* (JC14204), were upregulated after GA_3_ treatment in the present study. The exogenous CTK could also promote the DEGs related to JA-signaling transduction pathway in *J. curcas* [34]. It has been reported that JA play an important role in pollen development process [41], which might be a reason that JA content was upregulated both in GA and CTK treatment. Interestingly, exogenous cytokinin could increase endogenous GA and CTK transcription in previous study [34], but exogenous gibberellins promoted the endogenous CTK signaling and repressed the GA production in this study (Figure 6c and Figure 7a). It indicated that both CTK and GA were necessary for floral differentiation in *J. curcas*. The consistent results were that the floral transition was mediated by coordinate regulation of CTK and GAs activities in different species [42,43,44]. Furthermore, exogenous CTKs induced amounts of female flowers [5,7], but exogenous GAs just increased about 2-fold female flowers in the present study. Therefore, we suggested that the ratio of CTK and GA might significantly affect the female floral differentiation. The high percentage of CTK to GA may be appropriate for improving the female flower and increasing the fruit yield, and vice versa. A further study could focus on finding the best proportion of CTKs and GAs to obtain a high fruit yield in *J. curcas*.

Our previous study found that male floral initiation was associated with the flavonoid biosynthesis pathway, while female floral initiation was related to the phytohormone signal transduction pathway [30]. We combined the 951 DEGs obtained in this study with the previous DEGs dataset (GSE102894) detected in male or female floral initiation and development stages, respectively (Figure S2). The consistent results were that the co-detected DEGs in the libraries of the male floral initiation stage and GA_3_ treatment were also significantly enriched in flavonoid biosynthesis pathways, while the co-detected DEGs in the libraries of female floral differentiation and GA_3_ treatment were also related to the phytohormone signal transduction pathways (Figures S3 and S4). In previous studies, flavonoid biosynthesis inhibition resulted in the male sterility in Petunia [45], but not in *Arabidopsis thaliana* [46]. This suggested that flavonoids might act various roles in different species. Thus, a further study could focus on exploring the *Jatropha* flavonoid pathways to uncover the regulatory events associated with male floral differentiation. Additionally, the DEGs after exogenous CTK treatment were also significantly associated with phytohormone-signaling transduction pathways in *J. curcas* [19,34]. It indicated that the effects of phytohormone, which was induced by exogenous plant growth regulator, were mainly acted on female floral differentiation process in *J. curcas*.

## 4. Materials and Methods

### 4.1. Plant Material and GA Treatment

Three-year-old *Jatropha* clone was selected as the experimental material, which was planted in a field with latosolic red soil at a forestry trial base of South China Agricultural University (23.24° N, 113.64° E). The general climate has an average temperature of 20–22 °C and annual rainfall of 1720 mm in the rainy season from April to June. In our previous study, we found that spraying 40 mg/L GA_3_ onto the inflorescence meristems of *J. curcas* could significantly increase the flower number and the female-to-male flower ratio [8]. In the present study, the inflorescence meristems (about 0.5 cm in diameter) were sprayed with 5 mL GA_3_ working solution (40 mg/L) containing 0.05% (*v*/*v*) Tween-20, and the control inflorescence meristems were sprayed with distilled water containing 0.05% (*v*/*v*) Tween-20. For the RNA-seq analysis, the mixed samples of three inflorescence meristems were collected at 0, 6, 12, 24, and 48-h after the GA_3_ and control treatment, respectively. Two biological replicates were performed for each time point as in other studies [47,48,49]. All of the samples were immediately frozen in liquid nitrogen and stored at −80 °C until further used for RNA extraction. Additionally, fifteen inflorescence meristems of the GA_3_ and control treatment were used to investigate the effects of GA_3_ on branching of inflorescence in *J. curcas*. The experiments were carried out in September 2016, and all of the GA_3_ and control treatments were performed between 8:00 a.m. and 9:00 a.m.

### 4.2. Total RNA Extraction and Transcriptome Sequencing Analysis

Total RNA of each sample was extracted separately using TIANDZ Plant RNA Kit (TIANDZ column type RNAout 2.0, Beijing, China) following the instructions of the manufacturer. The quality of total RNA was investigated by agarose gel electrophoresis and Nanodrop 2100 (Thermo Scientific, Massachusetts, MA, USA). The eighteen cDNA libraries in this study, including T0_1, T0_2, CK6_1, CK6_2, CK12_1, CK12_2, CK24_1, CK24_2, CK48_1, CK48_2, T6_1, T6_2, T12_1, T12_2, T24_1, T24_2, T48_1, and T48_2, were constructed and sequenced using the Illumina HiSeq™ 4000 (Illumina, Santiago, CA, USA) platform at Novegene Bioinformatics Technology Co. Ltd. (Beijing, China). Sequence adaptors, N’s more than 10% and low-quality reads (Qphred ≤ 20 for >50% read) were removed [50]. When the quality control finished, the clean data was mapped to the genome sequence in NCBI for annotation (http://www.ncbi.nlm.nih.gov/genome/?term=Jatropha%20curcas). The TopHat v2.0.12 software (http://ccb.jhu.edu/software/tophat/index.shtml, 24 June 2014) was selected to map the reads producing in present study [51]. Both known and novel transcripts from TopHat alignment results were constructed and identified by the Cufflinks v2.1.1 (http://cole-trapnell-lab.github.io/cufflinks/releases/v2.1.1/, 4 November 2013) Reference Annotation Based Transcript (RABT) assembly method [52]. The alternative splicing (AS) events were clustered into 12 basic types by rMATS v3.0.8 software (http://rnaseq-mats.sourceforge.net/, 26 August 2013) [53]. The number of AS events in each sample was investigated, respectively. We used the following parameters to identify reliable novel genes: the length of transcript is more than 200 bp and two exons were detected at least in the transcript. The expression abundance of each read was calculated and converted to the expected number of fragments per kilobase of transcript sequence per million base pairs sequenced (FPKM) [54].

### 4.3. Identification and Annotation of DEGs

All DEGs were divided into 4 comparisons (T6 vs. CK6, T12 vs. CK12, T24 vs. CK24, T48 vs. CK48). Differential expression analysis of each comparison was performed using the DEGSeq R package (v.1.30.0, http://www-huber.embl.de/users/anders/DESeq/, 21 June 2012). Fold change (FC) is the gene expression difference between different samples. We used the threshold |log_2_(FC)| > 1 and *Q* < 0.005 as the criteria for identifying the DEGs. All of the upregulated or downregulated genes in the following description were regulated in the first comparison component. Functional enrichment and classification of DEGs were performed according to the Gene Ontology database (GO, http://www.geneontology.org/) and the Kyoto Encyclopedia of Genes and Genomes database (KEGG, http://www.kegg.jp/). GOSeq (http://bioinf.wehi.edu.au/software/goseq/, 4 February 2010) and KOBAS v2.0 software (http://genome.cbi.pku.edu.cn/download.html, 7 April 2005) were used to estimate the statistical enrichment of DEGs in GO terms and KEGG pathways, respectively [55,56]. Corrected *p*-value of 0.05 was set as the threshold for the significantly enrichment of GO terms and KEGG pathways. Transcription factor prediction analysis was carried out by iTAK v1.5 software (http://itak.feilab.net/cgi-bin/itak/index.cgi, 13 August 2012) [57]. The Blastx alignment was conducted between the DEGs and TAIR10 protein database (https://www.arabidopsis.org/). The heat maps in present study were performed by R software (v.2.15.3, https://cran.r-project.org/src/base/R-2/, 1 March 2013) [58].

### 4.4. Quantitative Real-Time PCR Validation

To validate the results of transcriptome, a total of 48 DEGs were selected for quantitative real-time PCR (qRT-PCR) analysis using the same plant materials used for RNA sequencing. The mixed samples of three inflorescence meristems were conducted for the DEG validation at different time points after the GA_3_ and control treatment. In addition, two biological replicates were carried out for each time point with three technological replications for each gene. These genes were selected because of their important function after GA_3_ treatment according to DEGs analysis in present study. The cDNA synthesis was conducted using PrimeScript^®^ II first Strand cDNA Synthesis Kit (TaKaRa, Kyoto, Japan). The specific primers were designed by Primer Premier 5.0 (PREMIER Biosoft, Palo Alto, CA, USA) (Table S3). qRT–PCR was performed on Roche LightCyler480 system (Roche, Basel, Switzerland) with SYBR Premix Ex Taq^TM^II (TaKaRa) [30]. The qRT-PCR procedure was as follows: 2 μL of cDNA dilution in H_2_O (about 50 ng/μL) was added to 10 μL of 2× SYBR^®^ buffer, with 1 μL (10 μM) of each primer and H_2_O to a final volume of 20 μL. The cycling reaction was 94 °C for 2 min, followed by 40 cycles of 94 °C for 10 s, 55 °C for 10 s and 72 °C for 20 s. *JcGAPDH* and *β-actin* (*Jcactin*) were used as internal controls [59]. The 2^−ΔΔ*C*t^ method was used to calculate the relative expression level of the DEGs [60]. 

## 5. Conclusions

The present study performed the transcriptional response analysis of the inflorescence meristems in *Jatropha curcas* following gibberellin treatment. Our results showed that 6.27–10.46 G clean data were obtained in GA_3_ treated and untreated samples, respectively. 951 DEGs were isolated at the different time points, compared with control samples. The 6-h time point was an important phase in the response to exogenous gibberellins in the present study. In addition, 8532 novel transcripts were found in present RNA-seq analysis, which could contribute to obtain new genes and exons related to inflorescence meristem differentiation in *J. curcas*. Furthermore, the plant endogenous IAA, ETH, ABA, and BR signaling were repressed after exogenous gibberellins treatment. The exogenous GA could also inhibit the endogenous GA biosynthesis and signaling pathways. However, the DEGs associated with a JA signal transduction pathway were upregulated following GA_3_ treatment to contribute to pollen development process. Both CTK and GA might be necessary for floral differentiation in *J. curcas*; the ratio of CTK to GA significantly affected the female floral differentiation and fruit yield. Moreover, the floral meristem determinacy genes (*JcLFY* and *JcSOC1*) and floral organ identity genes (*JcAP3*, *JcPI*, and *JcSEP1-3*) were significantly upregulated, but their negative regulator (*JcSVP*) was downregulated after GA_3_ treatment. Additionally, the effects of phytohormone, which was induced by exogenous plant growth regulator, mainly acted on female floral differentiation process in *J. curcas*.

This data will create a reference transcriptome for the genomics database of *J. curcas* for future studies.Our study will contribute to understanding the underlying transcriptional response mechanism of floral differentiation following GA_3_ treatment. We expect that the dataset will serve as a foundation to study the genes’ functions, which help in engineering high-yielding varieties in *J. curcas*.

## Figures and Tables

**Table 1:** The annotation of the 23 DEGs co-detected in the four comparisons after GA_3_ treatment.

Gene ID	*At.* locus	*At.* name	Blastx to TAIR10 Database
JC06413	AT5G13930.1	*TT4*	Flavonoid biosynthetic process
JC06540	AT3G51240.1	*TT6*	Flavonoid biosynthetic process
JC22745	AT5G07990.1	*TT7*	Flavonoid biosynthetic process
JC26359	AT5G61850.1	*LFY*, *LFY3*	Floral meristem determinacy
JC25970	AT1G20920.1	*RCF1*	mRNA splicing via spliceosome
JC02534	AT4G24340.1	Phosphorylase superfamily protein	Nucleoside metabolic process
JC08881	AT4G24340.1	Phosphorylase superfamily protein	Nucleoside metabolic process
JC08882	AT4G24340.2	Phosphorylase superfamily protein	Nucleoside metabolic process
JC08884	AT4G24340.3	Phosphorylase superfamily protein	Nucleoside metabolic process
JC08888	AT4G24340.4	Phosphorylase superfamily protein	Nucleoside metabolic process
JC16054	AT5G48810.1	*CB5-D*	Oxidation-reduction process
JC15089	AT3G01680.1	Contains interpro domain/s	Phloem development
JC04718	AT1G70830.1	MLP-like protein 28	Response to biotic stimulus
JC15401	AT2G45030.1	*EFG*/*EF2*	Translation elongation factor
JC26020	AT5G24090.1	*CHIA*	Response to light intensity
JC10078	AT1G49320.1	*USPL1*	Seed development
JC08649	AT5G43580.1	*UPI*	Serine protease inhibitor
JC04717	AT2G01520.1	MLP-like protein 328	Vegetative to reproductive phase transition
JC13242	AT1G70830.1	MLP-like protein 28	Defense response
JC10079	AT3G29075.1	Glycine-rich protein	-
JC12650	-	-	-
Novel00596	-	-	-
Novel00988	-	-	-

All of the genes were annotated with TAIR10 database. Gene ID is the gene number in RNA-seq database of *Jatropha curcas*. *At.* locus is the locus of homologous gene in *Arabidopsis thaliana*, *At.* name is the gene name of homologous gene in *Arabidopsis thaliana*. “-” indicated that no homologous genes was detected in TAIR10 protein database.

**Table 2:** The DEGs associated with GA biosynthetic and signaling pathways after GA_3_ treatment.

Gene ID	*At.* locus	*At.* name	Blastx to TAIR10 Database
JC04894	AT4G25420.1	*GA20OX1*	Gibberellin biosynthetic process
JC04895	AT4G25420.2	*GA20OX2*	Gibberellin biosynthetic process
JC01251	AT1G30040.1	*GA2OX2*	Gibberellin oxidation-reduction process
JC04619	AT1G30040.1	*GA2OX2*	Gibberellin oxidation-reduction process
JC18588	AT4G21200.1	*GA2OX8*	Gibberellin oxidation-reduction process
JC21273	AT4G21200.1	*GA2OX8*	Gibberellin oxidation-reduction process
JC22004	AT3G63010.1	*GID1B*	Positive regulation of gibberellin mediated signaling pathway
JC23405	AT5G27320.1	*GID1C*	Positive regulation of gibberellin mediated signaling pathway
JC20657	AT1G66350.1	*RGL1*	Gibberellic acid mediated signaling pathway
JC01849	AT3G50950.2	*ZAR1*	Gibberellic acid signal transduction
JC23160	AT1G09530.1	*PAP3*	Gibberellic acid mediated signaling pathway

**Table 3:** DEGs related to the other plant hormone-signaling transduction pathways after GA_3_ treatment.

Gene ID	*At.* locus	*At.* name	Blastx to TAIR10 Database
JC08238	AT1G19850.1	*ARF5*	Auxin-activated signaling pathway
JC06232	AT4G14550.1	*IAA14*	Auxin-activated signaling pathway
JC21840	AT4G14550.1	*IAA14*	Auxin-activated signaling pathway
JC23499	AT5G43700.1	*IAA4*	Auxin-activated signaling pathway
JC00759	AT5G54510.1	*GH3.6*	Auxin-activated signaling pathway
JC02272	AT4G14550.1	*IAA14*	Auxin-activated signaling pathway
JC14244	AT3G61830.1	*ARF18*	Auxin-activated signaling pathway
JC16280	AT2G14960.1	*GH3.2*	Response to auxin
JC19628	AT2G21220.1	*SAUR12*	Response to auxin
JC23402	AT3G21510.1	*AHP1*	Cytokinin-activated signaling pathway
JC17975	AT1G27320.1	*HK3*	Cytokinin-activated signaling pathway
JC14204	AT4G17880.1	*MYC4*	Jasmonic acid-activated signaling pathway
JC12669	AT1G32640.1	*MYC2*	Jasmonic acid-activated signaling pathway
JC23114	AT5G13220.1	*JAZ10*	Negative regulation of JA signaling pathway
JC13342	AT2G25490.1	*EBF1*	Negative regulation of ethylene-activated signaling pathway
JC07165	AT2G25490.1	*EBF1,*	Negative regulation of ethylene-activated signaling pathway
JC17700	AT3G23240.1	*ERF1,*	Ethylene-activated signaling pathway
JC14274	AT1G01360.1	*PYL9*	Abscisic acid-activated signaling pathway
JC02934	AT1G07430.1	*HAI2*	Negative regulation of ABA signaling pathway
JC22124	AT4G34160.1	*CYCD3*	Response to brassinosteroid

**Table 4:** The DEGs associated with MADS-box transcription factors after GA_3_ treatment in *J. curcas.*

Gene ID	*At.* locus	*At.* name	Blastx to TAIR10 Database
JC15882	AT2G22540.1	*SVP*, *AGL22*	Floral meristem determinacy
Novel00351	AT2G22540.1	*SVP*, *AGL22*	Floral meristem determinacy
JC26359	AT5G61850.1	*LFY*, *LFY3*	Floral meristem determinacy
JC11754	AT5G15800.1	*SEP1*, *AGL2*	Floral meristem differentiation
JC25593	AT5G15800.1	*SEP2*, *AGL2*	Floral meristem differentiation
JC17987	AT1G24260.1	*SEP3*, *AGL9*	Floral meristem differentiation
JC16067	AT1G22130.1	*AGL104*	Pollen maturation
JC14482	AT2G45660.1	*SOC1*, *AGL20*	Positive regulation of flower development
JC04507	AT3G54340.1	*AP3*	Petal and stamen development
JC13660	AT3G54340.1	*AP3*	Petal and stamen development
JC12153	AT5G20240.1	*PI*	Petal identity

## Supplementary Materials

Supplementary materials can be found at http://www.mdpi.com/1422-0067/19/2/432/s1.
