# Supplementary material for: Analysis of Transcriptional Responses of the Inflorescence Meristems in Jatropha curcas Following Gibberellin Treatment
Source: Int J Mol Sci. 2018 Feb 1;19(2):432. doi: 10.3390/ijms19020432 (PMC5855654; doi:10.3390/ijms19020432)
Supplement: Supplementary file 1 [file ijms-19-00432-s001.zip › Supplementary figures.pdf]

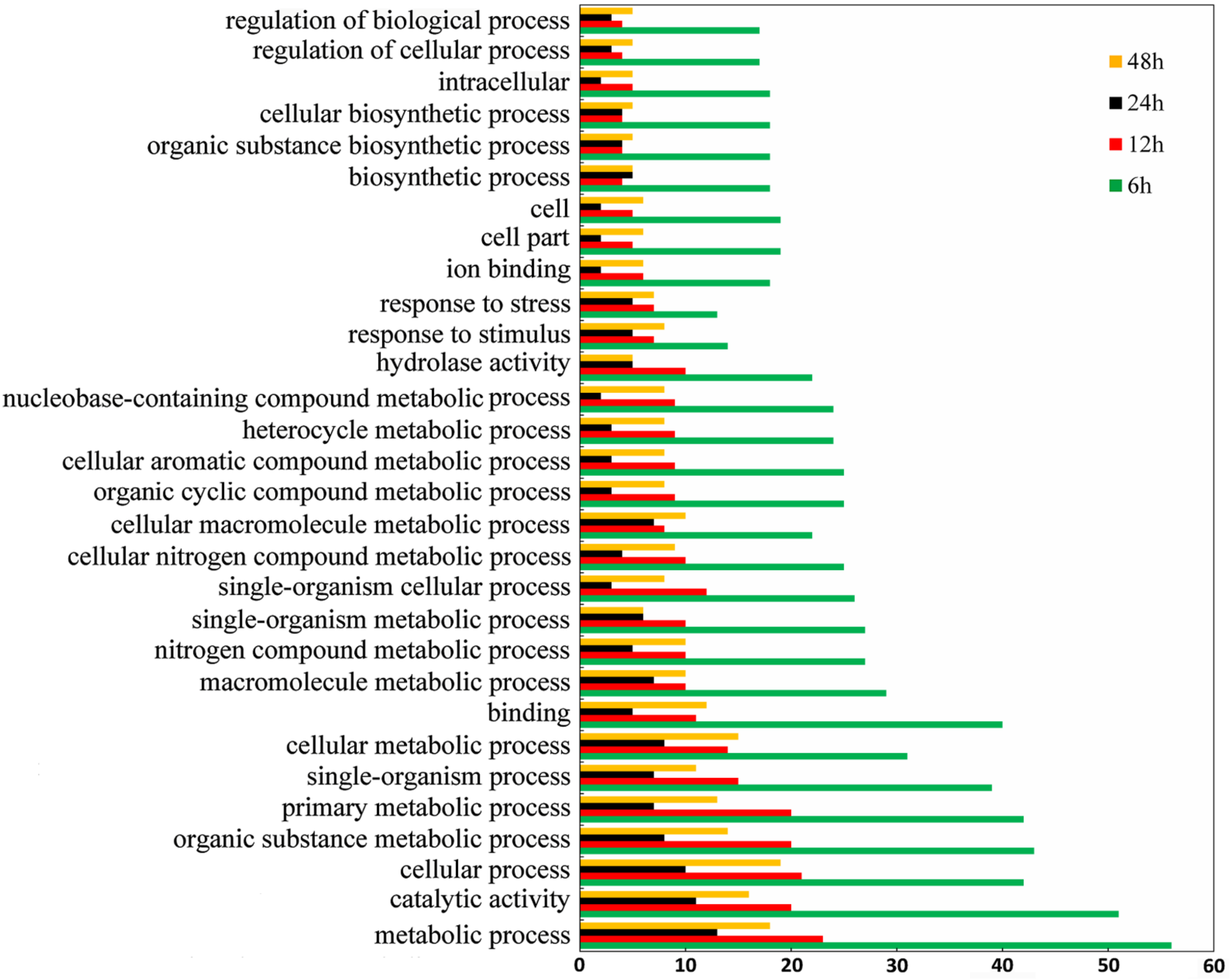

Figure S1. The top 30 comparative analysis of Gene Ontology category of DEGs at different time points after GA_3 treatment in *J. curcas*.

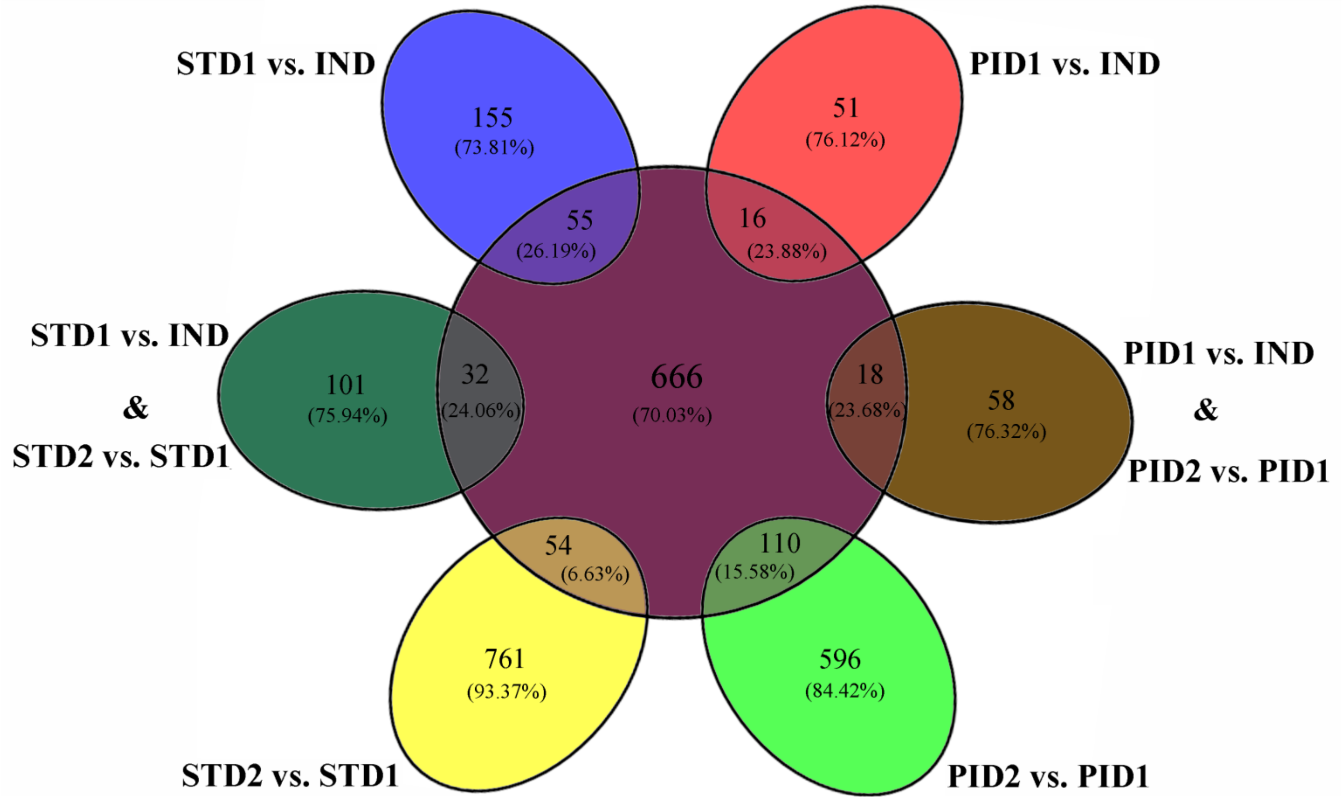

Figure S2 The venny diagram shared with numbers and proportion of DEGs detected in floral sex differentiation process and GA_3 treatment in *J. curcas*. The middle round area was the 951 DEGs detected in different time points after GA_3 treatment. The six ellipses were the DEGs exclusively detected in different comparisons associated with floral sex differentiation process, including STD1 vs. IND, STD1 vs. IND & STD2 vs. STD1, STD2 vs. STD1, PID1 vs. IND, PID1 vs. IND & PID2 vs. PID1, PID2 vs. PID1, respectively. IND was the stage of inflorescence meristems, STD1was the stage of male floral initiation, STD2 was the stage of ten complete stamens formed, PID1 was the stage of female floral initiation, PID2 was the stage of complete carpel and ovary formed. STD1 vs. IND & STD2 vs. STD1 were the DEGs exclusively co-detected in male floral differentiation process, and PID1 vs. IND & PID2 vs. PID1 were the DEGs exclusively co-detected in female floral differentiation process.

(a)

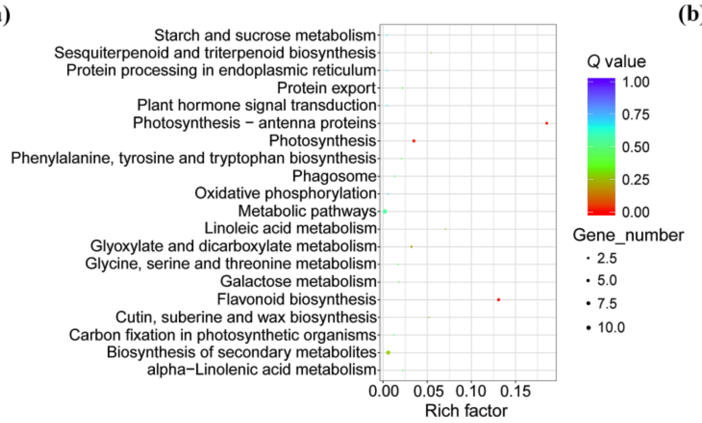

(b)

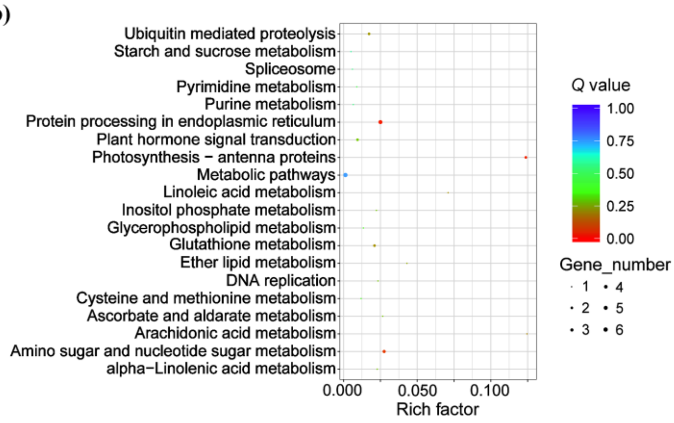

Figure S3 The KEGG enrichment pathways involved in male floral differentiation after GA_3 treatment in *J. curcas*. The Rich factor indicated the percentages of DEGs belong to the corresponding pathway. The left y-axis represented the enrichment pathways. The sizes of bubble represent the number of DEGs in the corresponding pathway, and the colors of the bubble represent the enrichment Q value of the corresponding pathway. (a) KEGG enrichment pathways of the DEGs co-detected in STD1 vs. IND and GA_3 treatment. (b) KEGG enrichment pathways of the DEGs co-detected in STD1 vs. IND & STD2 vs. STD1 and GA_3 treatment, STD2 vs. STD1 and GA_3 treatment.

(a)

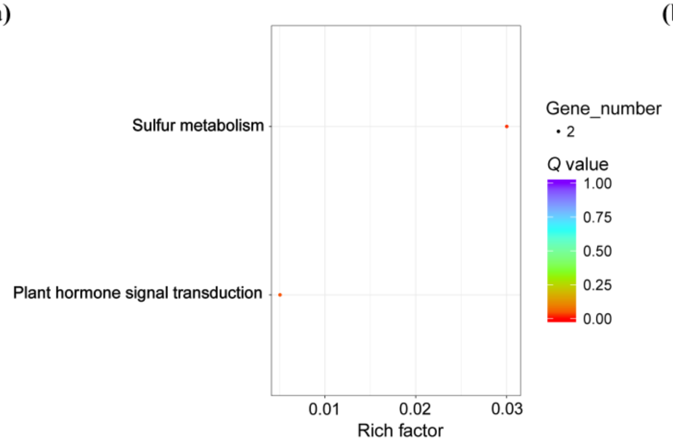

(b)

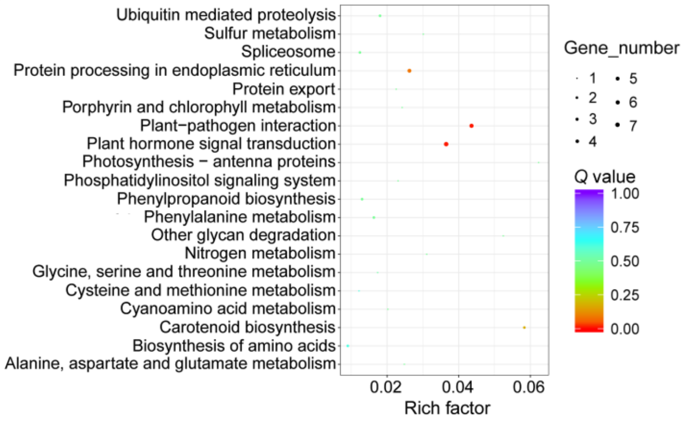

Figure S4 The KEGG enrichment pathways involved in female floral differentiation after GA_3treatment in *J. curcas*. The Rich factor indicated the percentages of DEGs belong to the corresponding pathway. The left y-axis represented the enrichment pathways. The sizes of bubble represent the number of DEGs in the corresponding pathway, and the colors of the bubble represent the enrichment Q value of the corresponding pathway. (a) KEGG enrichment pathways of the DEGs co-detected in PID1 vs. IND and GA_3 treatment. (b) KEGG enrichment pathways of the DEGs co-detected in PID1 vs. IND & PID2 vs. PID1 and GA_3 treatment, PID2 vs. PID1 and GA_3 treatment.
